# Supplementary material for: Cloud BioLinux: pre-configured and on-demand bioinformatics computing for the genomics community
Source: BMC Bioinformatics. 2012 Mar 19;13:42. doi: 10.1186/1471-2105-13-42 (PMC3372431; doi:10.1186/1471-2105-13-42)
Supplement: Additional file 1 — Supplementary 1 Cloud BioLinux software documentation in the form of a mini, self-contained website. Users need to download and uncompress the .zip file, and open through a web browser the "index.html" file available on the main directory. (ZIP 1823 kb). [file 1471-2105-13-42-S1.ZIP › Cloud-BioLinux-Package-Documentation/docs/nucmer.html]

Bio-Linux Software Documentation Pages

Back to search form

## nucmer

|  |  |
| --- | --- |
| Name | nucmer |
| Description | **nucmer** is a part of the MUMmer package, for the rapid alignment of very large DNA and amino acid sequences.  **nucmer** (NUCleotide MUMmer) is the most user-friendly alignment script for standard DNA sequence alignment. It is a robust pipeline that allows for multiple reference and multiple query sequences to be aligned in a many vs. many fashion.  **References:**  Delcher AL, Kasif S, Fleischmann RD, Peterson J, White O, Salzberg SL: Alignment of whole genomes, Nucleic Acids Res. 1999 Jun 1;27(11):2369-76.[Entrez]    Delcher AL, Phillippy A, Carlton J, Salzberg SL: Fast algorithms for large-scale genome alignment and comparison, Nucleic Acids Res. 2002 Jun 1;30(11):2478-83.[Entrez]    Kurtz S, Phillippy A, Delcher AL, Smoot M, Shumway M, Antonescu C, Salzberg SL: Versatile and open software for comparing large genomes, Genome Biol. 2004;5(2):R12. Epub 2004 Jan 30.[Entrez] |
| Homepage | http://www.tigr.org/software/mummer/ |
| Remote Documentation | http://www.tigr.org/software/mummer/manual/ |
